# Cap-Independent Translation Promotes *C. elegans* Germ Cell Apoptosis through Apaf-1/CED-4 in a Caspase-Dependent Mechanism

**DOI:** 10.1371/journal.pone.0024444

**Published:** 2011-09-01

**Authors:** Vince Contreras, Andrew J. Friday, J. Kaitlin Morrison, Enhui Hao, Brett D. Keiper

**Affiliations:** Department of Biochemistry and Molecular Biology, Brody School of Medicine at East Carolina University, Greenville, North Carolina, United States of America; University of Washington, United States of America

## Abstract

Apoptosis is a natural process during animal development for the programmed removal of superfluous cells. During apoptosis general protein synthesis is reduced, but the synthesis of cell death proteins is enhanced. Selective translation has been attributed to modification of the protein synthesis machinery to disrupt cap-dependent mRNA translation and induce a cap-independent mechanism. We have previously shown that disruption of the balance between cap-dependent and cap-independent *C. elegans* eIF4G isoforms (IFG-1 p170 and p130) by RNA interference promotes apoptosis in developing oocytes. Germ cell apoptosis was accompanied by the appearance of the Apaf-1 homolog, CED-4. Here we show that IFG-1 p170 is a native substrate of the worm executioner caspase, CED-3, just as mammalian eIF4GI is cleaved by caspase-3. Loss of Bcl-2 function (*ced-9ts*) in worms induced p170 cleavage *in vivo*, coincident with extensive germ cell apoptosis. Truncation of IFG-1 occurred at a single site that separates the cap-binding and ribosome-associated domains. Site-directed mutagenesis indicated that CED-3 processes IFG-1 at a non-canonical motif, TTTD^456^. Coincidentally, the recognition site was located 65 amino acids downstream of the newly mapped IFG-1 p130 start site suggesting that both forms support cap-independent initiation. Genetic evidence confirmed that apoptosis induced by loss of *ifg-1* p170 mRNA was caspase (*ced-3*) and apoptosome (*ced-4*/Apaf-1) dependent. These findings support a new paradigm in which modal changes in protein synthesis act as a physiological signal to initiate cell death, rather than occur merely as downstream consequences of the apoptotic event.

## Introduction

During programmed cell death (apoptosis), cells commit to the systematic disassembly of their metabolic framework. Recent evidence suggests that signaling requires prominent translational control mechanisms for both the commitment and execution phases of cell death [1]. *De novo* protein synthesis decreases initially in apoptosing cells, particularly from growth-related mRNAs. Apoptosing cells nevertheless upregulate the synthesis of several “death-related” proteins through an alternative mode of translation initiation. The switch in protein synthesis affords rapid responses to various types of stressors, allowing the cell to recover from injury, or submit to a path of suicide [1].

Apoptosis affects cellular mRNA translation primarily at initiation, more specifically at the mRNA binding step. Recruitment of mRNAs for translation usually involves a 5′ cap-mediated scanning mechanism. The eukaryotic translation initiation factor 4 (eIF4) complex catalyzes the joining of mRNA to ribosomes. The basic complex (eIF4F) is comprised of eIF4E, which binds the 7-methylguanosine cap, eIF4A, an mRNA helicase, and eIF4G, a scaffold protein that coordinates these factors and bridges the interaction between the mRNA and the 40S ribosomal subunit [2], [3]. During apoptosis proteolytic enzymes called caspases induce a signaling cascade that results in cleavage of several translation initiation proteins including eIF2 alpha and eIF4G [4], [5]. Disruption of the eIF4F complex leads to not only the attenuation of global protein synthesis, but also the selective synthesis of death proteins. Cleavage of eIF4GI has been shown to enhance translation of the apoptotic peptidase-activating factor 1 (Apaf-1, an apoptosome subunit), and the “death associated protein” p97/DAP5 [6]. The cap- and poly(A)-associating N-terminal domain is removed from the RNA/ribosome-binding central domain. The latter complex still catalyzes initiation by a cap-independent mechanism. Thus, translation of mRNAs required for rapid responses to stress depends on the translational competence of eIF4F factors like eIF4G.

**Figure 1:**
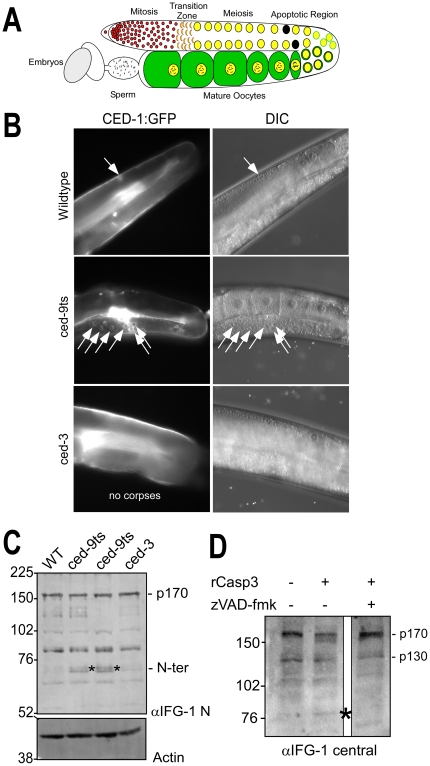
IFG-1 is cleaved during *C. elegans* apoptosis *in vivo* and by human caspase-3. (A) Diagram depicting one lobe of the adult gonad. Germ cells begin as a stem cell population of mitotically dividing cells in a common cytoplasm that transitions into a meiotic program. As oocytes mature they become fully cellularized and accumulate cytoplasmic components and condensed bivalent chromosomes prior to fertilization. The region of naturally occurring apoptotic cell death is indicated. (B) Fluorescence images depicting germ cell apoptotic events induced by loss of *ced-9* function. One lobe of the gonad from adult wild type, temperature-sensitive *ced-9ts* (n1653), and *ced-3* (n2452) strains expressing the apoptotic marker CED-1::GFP are shown. GFP-positive apoptotic corpses are designated by white arrows. Overlapping DIC images to the right demonstrate normal gonad and oocyte morphologies. In the wild type and *ced-3* panels, the path of the intestine has obscured fully grown oocytes in the proximal arm, but younger oocyte nuclei are visible in the distal arm prior to the bend, in the region where germ cell apoptosis occurs. (C) Western blot of IFG-1 p170 cleavage *in vivo* in *ced-9(ts)* adult worms. Worms treated 48h at 25°C were homogenized and the extract analyzed by 8% SDS-PAGE. Two independent lines of *ced-9ts* (n1653) worms were analyzed. Immunoblotting was performed using IFG-1 N-terminal and actin antibodies. N-terminal cleavage products are indicated (*). (D) Immunoblot analysis demonstrating *ex vivo* cleavage of IFG-1 p170 from total wild type *C. elegans* lysate by purified recombinant human caspase-3 (Sigma). The blot was probed with an IFG-1 central domain antibody that detects both p170 and p130 isoforms. The migration of cleavage products is indicated (*).

Multiple isoforms of eIF4G are encoded by three separate genes in mammals [7]. Full length eIF4GI and eIF4GII are both expressed broadly in tissues and are cap-dependent (capable of establishing eIF4F complexes with eIF4E). The shorter p97/DAP5 isoform, however, lacks the N-terminus and catalyzes cap-independent initiation (establishes eIF4F complexes without eIF4E). All three eIF4G proteins (I, II, and p97) are proteolytically processed by caspases. Caspase-3 cleaves eIF4GI into three distinct fragments by recognizing the sites DLLD^532^ and DRLD^1176^ [8], [9]. The p97 isoform is likewise processed into a smaller p86 fragment (Fig. 1A). Despite their inability to associate with the mRNA cap, the cleaved products still participate in protein synthesis and are found in polyribosomal complexes [10], [11]. Both have been implicated in the stimulation of internal ribosome entry site (IRES)-mediated translation during cell death [6], [10], [12], [13]. IRES elements are found in mRNAs encoding the apoptotic proteins Bcl-2, X-linked inhibitor of apoptosis protein (XIAP), p97, and Apaf-1 [14]. Therefore, loss of functional eIF4G domains from the initiation complex changes the mode of initiation and recruits a new type of mRNA for translation. The change in translation mode allows the cell to operate protein synthesis during all states of physiological stress (cell cycle arrest, attempts at repair, and suicide of the unsalvageable cell).

Apoptosis was first characterized in the simple worm *Caenorhabditis elegans*, and is marked by distinct morphological changes that designate a cell for destruction and removal [15], [16]. The germ line of *C. elegans* has long been a useful *in vivo* model for studying programmed cell death in a whole organ. The fate of cells undergoing apoptosis can be observed through each of the three distinct stages: specification of the dying fate, execution of cell death mechanisms, and finally recognition and engulfment of the dying cell [17]. Coordinated removal of select cells is critical for maintaining cellular homeostasis during both gametogenesis and embryonic development [18]. The *ced* (cell death abnormal) genes are responsible for all steps in execution and engulfment of cells fated to die in the worm [19]. Key regulators in the apoptotic pathway are the survival factor CED-9 (Bcl-2 homolog) the pro-apoptotic Apaf-1 homolog, CED-4, and the executioner caspase, CED-3 [20], [21], [22]. This conserved pathway used for natural cell death events in a whole organism provides an optimal context for studying protein synthesis mechanisms during apoptosis.

Our lab recently discovered that *C. elegans* eIF4G (IFG-1) isoforms promote alternative protein synthesis mechanisms and apoptotic selection during germ cell development [23]. Two major IFG-1 isoforms, p170 and p130, are encoded by a single gene (*ifg-1),* but differ in their ability to associate with mRNA cap complexes. The shorter IFG-1 p130 lacks the N-terminal eIF4E binding region and has been suggested to participate in cap-independent initiation, much like human p97. Disrupting the balance between p170 and p130 by RNA interference (RNAi) resulted in a dramatic increase in the number of oocytes undergoing apoptosis, upregulation of the Apaf-1 homolog CED-4, and the appearance of apoptosomes [23]. However, it was not determined how or even if the initiation of the caspase cascade resulted from this disruption. Here we report that propagation of the programmed cell death signal, as a result of IFG-1 isoform imbalance, requires a caspase-dependent mechanism and acts through the apoptosome factor Apaf-1/CED-4. Cap-independent synthesis was not sufficient to drive apoptosis in oocytes without the upstream signaling events. We further demonstrate that, like human eIF4G proteins, IFG-1 p170 is a substrate for the executioner caspase-3 homolog, CED-3. The findings suggest that a change in protein synthesis mechanism is an upstream signaling event rather than a later consequence of the apoptotic fate. The significance of eIF4G integrity, the balance of isoforms, and the protein synthetic mechanisms that cause apoptotic signaling in gamete development are discussed.

## Results

The germ line is organized as a linear array of proliferating, and then differentiating cells that share a common cytoplasm (syncytium) derived from a single stem cell progenitor [24]. Apoptosis is critical for the establishment of gonad architecture as well as maintenance of germ cell number and the robust growth of fertile oocytes/eggs. Over half of all potential oocytes are eliminated by apoptosis during meiotic growth in the gonad (Fig. 1A) [25]. It has been suggested that this death is a mechanism used to eliminate excess germ cell nuclei generated by the mitotic stem cells. These deaths occur in a defined region of the worm gonad that can be readily assayed by apoptotic markers. The eliminated cells in essence act as nurse cells by synthesizing cytoplasmic components (mRNAs, ribosomes, organelles) required by those oocytes that will survive to be fertilized. As their fate progresses toward apoptosis, cells destined to die (termed “corpses”) undergo characteristic biochemical and morphological changes and appear as highly refractive, button-like spheres in preparation for their removal from the syncytial wake [19]. Finally, somatic cells from the sheath surrounding the meiotic cells of the gonad recognize and remove the corpse via a scavenger receptor known as CED-1. In our studies, worm strains bearing a *ced-1::gfp* transgene allow easy recognition and counting of fluorescence-decorated corpses.

### IFG-1 is cleaved during CED-9-mediated germ cell apoptosis and is a caspase substrate

To test whether IFG-1 was targeted by the apoptotic pathway, we created two independent lines bearing a temperature-sensitive Bcl-2 mutation (*ced-9ts*) and the *ced-1::gfp* “corpse reporter”. These lines allowed us to induce extensive spontaneous germ cell apoptosis *in vivo* independent of *ifg-1* disruption. Growth of *ced-9ts* young adult worms at 25°C for 36h caused accumulation of numerous germ cell corpses decorated with fluorescence (Fig 1B). At this early time point, the effect on the integrity of the gonad remained mostly intact, as evidenced by the normal morphology of surrounding early and late-staged oocytes and the organ as a whole (Fig. 1B, DIC). Biochemical analysis of IFG-1 p170 by western blot, however, revealed that these worms also accumulated N-terminal cleavage products migrating at approximately 70 kDa (Fig. 1C), suggesting IFG-1 was targeted by a protease during apoptosis. Because neither all oocytes, nor all somatic cells entered apoptosis even in the *ced-9t*s worms, most IFG-1 p170 remained intact. IFG-1 cleavage was not caused by temperature shift *per se*, since these products were not abundant in wild type or in *ced-3* mutant worms that were similarly treated. The 70 kDa product also suggests a discrete, site-specific cleavage rather than general degradation, and the size suggests a site in the N-terminal domain, as has been observed for human eIF4GI [26], [27]. Thus, cleavage of *C. elegans* eIF4G is occurring during natural apoptotic events *in vivo*. The primary executioner caspase in worms is CED-3. How CED-3 contributes to the control of apoptosis in the germ line to date remains unclear because very little is known about its natural substrates in developing gametes [28].

In an initial attempt to determine whether the cleavage was actually due to an apoptotic caspase, we first assayed whether native *C. elegans* eIF4G (IFG-1) was a substrate for mammalian caspase-3. Protein extracts from whole worm lysates were incubated with 100 ng recombinant human caspase-3 (Sigma). Western blotting showed that IFG-1 p170 is depleted in the presence of caspase-3 that was blocked by the presence of the pan-caspase inhibitor, carbobenzoxy-valyl-alanyl-aspartyl-[O-methyl]- fluoromethylketone (z-VAD-fmk, Fig. 1D). Caspase-3 treatment generated a smaller central fragment of approximately 80 kDa. Because *C. elegans* IFG-1 protein is quite labile and subject to indirect proteolysis by worm or bacterial proteases, we were unable to detect the N-terminal fragment observed upon in vivo apoptotic induction (data not shown). Detection of the 80 kDa C-terminal fragment *in vitro* required use of numerous protease inhibitors, some of which may interfere with the activity of caspase-3. Based on its migration and use of a central domain antibody, this fragment is itself a secondary cleavage product, but gives evidence of IFG-1 p170 targeting by a known executioner caspase. Interestingly, there appeared to be little or no change in the levels of the smaller IFG-1 p130 isoform in the presence of caspase-3.

### IFG-1 is a substrate for CED-3 *in vitro*

**Figure 2:**
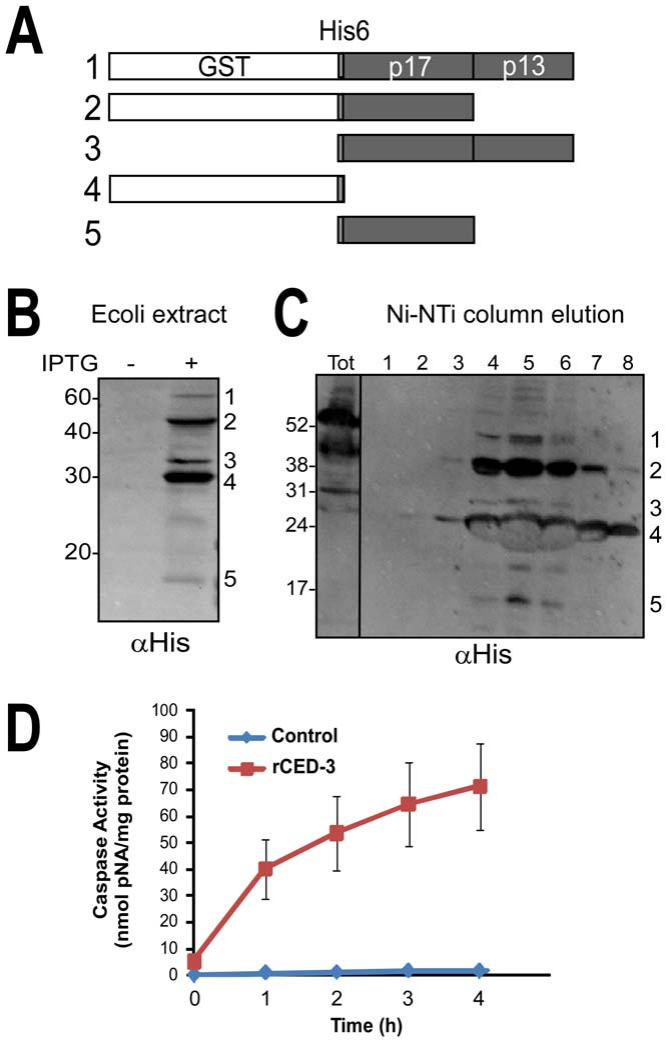
Generating catalytically active *C. elegans* recombinant CED-3. (A) Schematic depicting rCED-3 processed intermediates produced in E. coli. The full length (58 kDa) CED-3 consists of the catalytic amino acids 221–503 fused to GST and six His tags. Autocatalysis of rCED-3 into individual subunits (p17 and p13) that are required to create the mature heterotetramer are numbered 1–5. (B) rCED-3 expression was induced in E. coli using 100 µM IPTG and lysates analyzed by immunoblotting with an anti-His6 monoclonal antibody (Genscript). The mature p13 subunit contains no His6 tag and is therefore not detected. (C) The tagged proteins were subsequently purified by Ni-NTA affinity chromatography and elutions subjected to western blotting with His6 antibodies. (D) Caspase activity for rCED-3 was determined using chromogenic substrate Ac-DEVD-pNA (Promega) and measured at a wavelength of 405 nm. Partially purified enzyme (10 µl; 133–196 µg total protein) was added to the substrate (0.2 mM) and the rate of proteolysis monitored over a 4 h period. Activity (U) is expressed as nmoles pNA released per milligram protein per hour. Caspase activity (35 U/h) was compared to control reactions containing equivalent amounts of a bovine serum albumin (0.78 U/h), showing a 44-fold increase in rate. Data is representative of three independent preparations of rCED-3. Error bars are S.E.M.

The cleavage observed *in vivo* and in whole worm extracts could be due to indirect or secondary proteolytic activity rather than direct substrate cleavage by a defined caspase. These complications were overcome using recombinant IFG-1 and recombinant human and worm caspase-3. CED-3 is essential for the majority of developmentally associated apoptotic deaths in the worm [29], [30]. Like mammalian effector caspases, CED-3 is a highly specific protease that cleaves proteins at a tetrapeptide recognition sequence containing an aspartate residue in the P1 position [28]. CED-3 is synthesized as a catalytically inactive proenzyme of approximately 32 kDa [31]. Upon activation it is autoprocessed to form a heterotetramer composed of large subunit, p17 and small subunit p15/p13 (Fig. 2A). Given the similar substrate specificities for human caspase-3 and CED-3, we considered it likely that the worm caspase would cleave IFG-1 p170 and set out to demonstrate it empirically. This enzyme is unavailable commercially, so we generated active recombinant *C. elegans* CED-3 (rCED-3) using previously described purification conditions [28]. Sequences encoding catalytic portion (amino acids 221-503) were fused to N-terminal GST and 6X His tags and expressed in *E. coli*. Expression of the protein in bacteria revealed robust autocatalytic processing into well-defined subunits. Western blotting of bacterial lysate showed very little full length GST-His^6^-CED-3^201–503^, but considerable accumulation of processed intermediates (Fig. 2B). Similar rCED-3 intermediates, which include the p17 and p13 subunits required for activity, were co-purified upon Ni-NTA affinity chromatography (Fig. 2C). Proteolytic activity was confirmed in the partially purified rCED-3 using the synthetic tetrapeptide substrate Ac-DEVD-pNA, revealing a 53- fold increase in enzymatic rate above background (Fig. 2D). These data demonstrate that rCED-3 displays similar autocatalytic activity and substrate specificity to other purified effector caspases [31].

**Figure 3:**
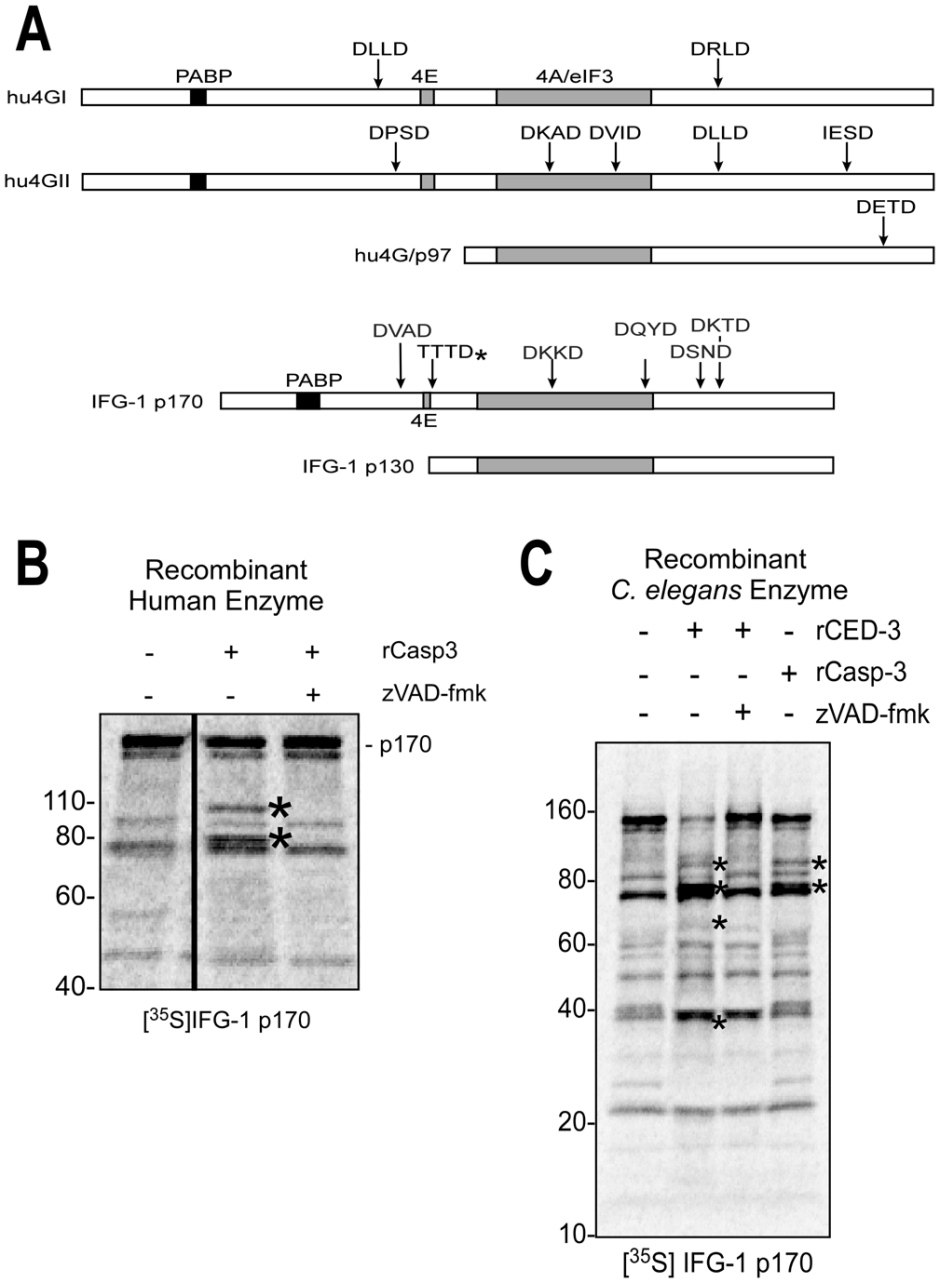
IFG-1 is a substrate for *C. elegans* CED-3. (A) Diagram depicting the known caspase-3 processed sites within human eIF4GI, eIF4GII, and p97 (upper three bars) and aligned IFG-1 p170 and p130 isoforms (lower bars) showing positions of predicted caspase sites (DXXD) as well as the actual, non-canonical *C. elegans* caspase (CED-3) cleavage site determined in this report (TTTD*). Relative positions of binding sites for major translation factor partners (PABP, eIF4E, and conserved eIF4A/eIF3 binding region) are also shown. (B) Cleavage of *in vitro* synthesized IFG-1 p170 by human caspase-3 (rCasp-3). Radiolabeled full length IFG-1 (1–1156) incubated with caspase-3 (2 h at 37°C). IFG-1 p170 produced *in vitro* migrated substantially smaller (∼150 kDa) than p170 detected in worm extracts by immunoblot. Aberrant migration is a common trait of eIF4Gs from most species, and may be due in part to posttranslational modifications *in vivo*. Smaller radiolabeled proteins in the untreated lane arise from internal translation starts *in vitro*. Two discreet cleavage fragments (*) were observed following caspase treatment. No processing of p170 was detected in the presence of the pan-caspase inhibitor z-VAD-fmk. (C) Recombinant [^35^S]-labeled full length IFG-1 (1–1156) was incubated with *C. elegans* rCED-3 under similar conditions as in (B); human caspase-3 cleavage products were also resolved for size comparison. Proteolysis by both caspase-1 and rCED-3 was inhibited using z-VAD-fmk. The caspase-3-specific inhibitor, ac-DEVD-cho, was also effective (not shown). Both primary and secondary processed fragments are marked (*).

**Figure 4:**
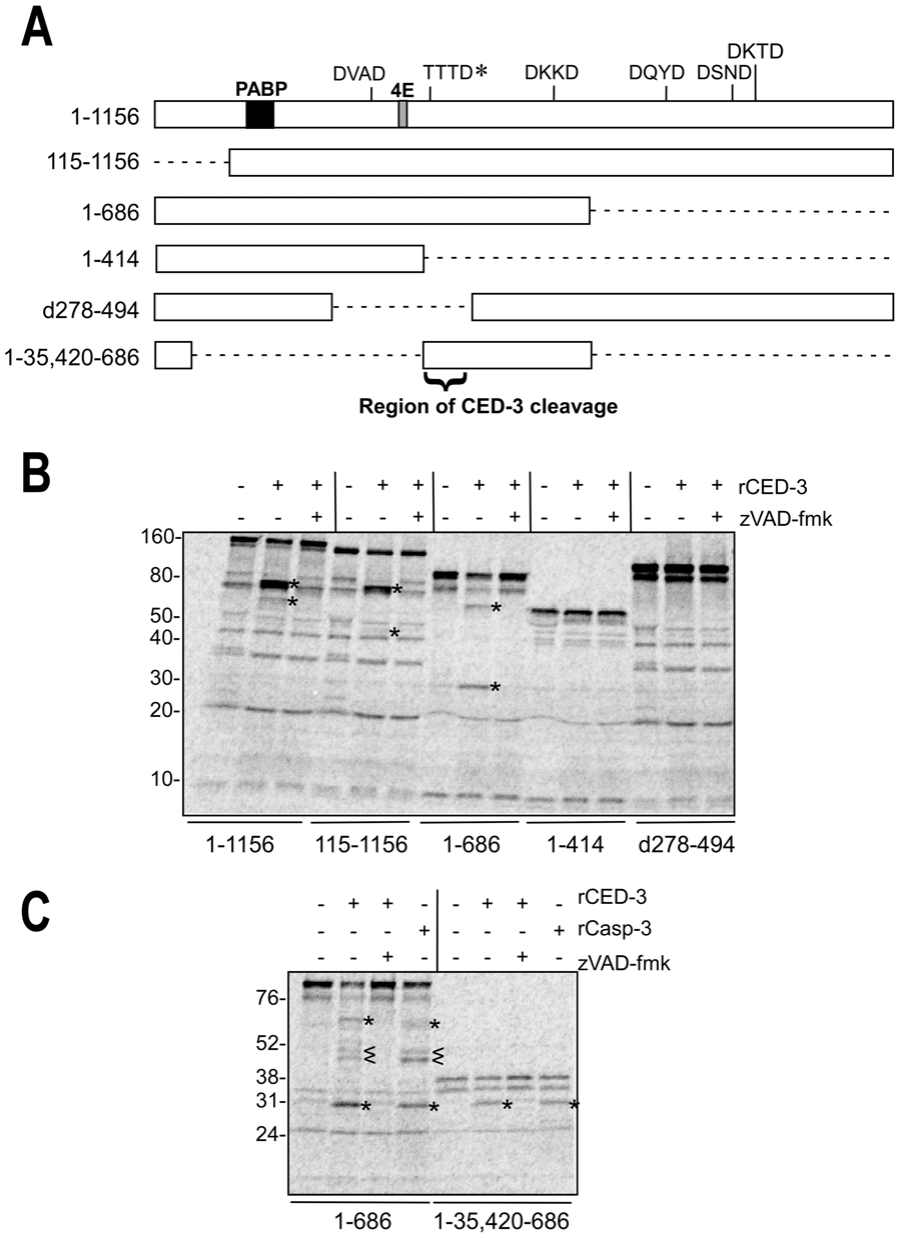
CED-3 cleaves IFG-1 downstream of the eIF4E binding site. (A) Schematic showing the series of IFG-1 truncated proteins (drawn to scale) used to delineate a region of CED-3 cleavage. Binding regions for other translation factors (PABP and eIF4E) within full length IFG-1 (1–1156) are indicated as well as caspase-3 consensus sites. The authentic CED-3 cleavage site determined in this report is marked by (*). Dashed lines indicate deleted portions of the peptides. (B) and (C) *In vitro* cleavage of IFG-1 truncation constructs by rCED-3 was carried as previously described in the presence or absence of z-VAD-fmk. [^35^S]-labeled products were resolved on either a 4–20% gradient (B) or 12% (C) SDS-PAGE gel and substrate cleavage detected by phosphorimaging. rCasp-3 (25 ng) was added to show comparable cleavage by the human effector caspase. Cleavage products are represented by (*). Secondary cleavage products are designated by (<).

Mammalian caspase-3 has been shown to recognize the consensus peptide DXXD motif in eIF4GI (2 sites) and p97 (1 site) that liberates central domain fragments called M-FAG and p86, respectively [32], Fig. 3A). To determine how these sites might correspond to potential sites in IFG-1, we conducted cleavage assays using both recombinant substrate and protease in a rabbit reticulocyte lysate (RRL). [^35^S]-labeled IFG-1 p170 (unmodified migrates at 150 kDa) was synthesized in RRL from a full-length cDNA and recombinant caspases added to assay direct proteolysis. Recombinant human caspase-3 (Sigma) directly cleaved *C. elegans* IFG-1 p170 in an extract devoid of any other *C. elegans* or *E. coli* proteins. Discrete products of approximately 95 and 80 kDa were generated by the human caspase (Fig. 3B). Addition of the inhibitor z-VAD-fmk completely prevented cleavage, indicating that the processed fragments were caspase-dependent. Control reactions containing bovine serum albumin failed to elicit any cleavage of IFG-1. These results show that IFG-1 p170 is a substrate for the human caspase-3, but do not address the enzyme natively found in the worm gonad. We next tested whether IFG-1 could be similarly targeted by the *C. elegans* main effector caspase, CED-3. Using rCED-3 we performed similar *in vitro* digestion of radiolabeled full length IFG-1 p170. Cleavage by the *C. elegans* caspase was very efficient, and two major fragments of approximately 95 and 80 kDa were observed representing N- and C-terminal cleavage products, respectively (Fig. 3C). However, our results indicate that under conditions of extensive cleavage the 95 kDa product likely gets further processed (Compare Fig. 3C and 4B). Secondary cleavage would also account for the accumulation of a smaller 70 kDa N-terminal intermediate *in vivo* (Fig. 1C). Cleavage of the 150 kDa full length IFG-1 and appearance of products was prevented in the presence of z-VAD-fmk, verifying that the cleavage of IFG-1 is solely due to the worm caspase. Furthermore, our rCED-3 was also able to cleave *in vitro* synthesized CED-9, a known anti-apoptotic substrate that had been previously described (data not shown) [20]. The major products resulting from IFG-1 p170 cleavage were similar to those generated by digestion with human caspase-3. These results showed that *C. elegans* CED-3 specifically cleaves IFG-1 p170. Direct caspase-mediated cleavage of non-mammalian eIF4G proteins has not previously been demonstrated, nor its effects on the protein synthetic programs that mediate physiological/developmental apoptosis.

### Determining rCED-3 cleavage sites in IFG-1

Cleavage of IFG-1 p170 by both recombinant human caspase-3 and *C. elegans* CED-3 suggested that a conserved eIF4G substrate recognition motif is shared among the effector caspases from species as divergent as mammals and nematodes (see Fig. 3A). The discrete product sizes infer that perhaps a single major cleavage site initially bifurcates the protein. Potential protease recognition sites for caspase-3 (DXXD) in IFG-1 were identified spanning the length of IFG-1 and include DVAD^334^, DKKD^671^, DQYD^784^, DSND^890^, and DKTD^931^ (Fig. 4A). We designed constructs to generate truncated *in vitro* synthesized [^35^S]-labeled IFG-1 proteins to systematically determine if any of these motifs were recognized by rCED-3 (Fig. 4B). Full length (1156 amino acid) IFG-1 is processed into 80 and 70 kDa fragments upon cleavage by rCED-3 (Fig. 4B, Lane 2). As stated above, the appearance of the 70 kDa fragment is likely due to secondary cleavage of the initial 95 kDa N-terminal product. Of the five potential caspase consensus sites, most occur near the C-terminal end of IFG-1. N-terminally truncated IFG 115–1156 and C-terminally truncated IFG 1-686 and 1-414 were incubated with rCED-3 to identify the region containing the major site of cleavage. IFG 115–1156 was cleaved into products of 80 kDa and 50 kDa respectively (Fig. 4B, Lane 5). The 80 kDa fragment migrated identically to the 80 kDa product from cleaved full length IFG 1–1156 (Fig. 4B, Lane 2). By contrast, no 70 kDa fragment was detected. Rather a smaller product (50 kDa) was observed and consistent with the absence of 114 N-terminal amino acids. The cleavage pattern therefore indicates that the 70 kDa fragment generated from full length IFG-1 is an N-terminal product. The 80 kDa fragment must be a C-terminal product, since the latter is present in both IFG 1–1156 and IFG 115–1156 reactions. The result further suggests that a more central cleavage site, rather than one near the C-terminus, is being used as the primary rCED-3 site.

The N-terminal domain of eIF4Gs causes the proteins to migrate aberrantly on SDS-PAGE due to the unique composition of amino acids within the hinge region [33]. This is also true of *C. elegans* IFG-1 since the protein has a predicted size of 129 kDa but migrates at either 150 kDa (*in vitro* synthesized) or approximately 170 kDa (worm extracts; Fig. 1). To optimize resolution of electrophoretic mobility and use fragment size to predict site placement, we analyzed rCED-3 cleavage of C-terminally truncated IFG-1 proteins (IFG 1-414 and IFG 1–686). Incubation with rCED-3 produced cleavage of IFG 1–686 only (Fig. 4B, Lane 8). Cleavage of IFG 1–686 generated major products corresponding to 70 kDa (N-terminal fragment) and 30 kDa (C-terminal fragment). Interestingly, the 70 kDa fragment comigrated with the 70 kDa band from IFG 1–1156 (Fig. 4B lanes 2 and 8), confirming that it is an N-terminal product. In contrast, IFG 1–414 was fully resistant to active rCED-3 (Fig. 4B, lanes 10–12), demonstrating that no cleavage site is present upstream of position 414. Based on these results the major rCED-3 cleavage site in IFG-1 p170 was determined to be between amino acids 414–686.

The data thus far indicate a major processing site for IFG-1 by rCED-3 located centrally in the protein (amino acids 414–686). Furthermore, radiolabeled IFG d278–494 that lacked 192 amino acids in the identified region was resistant to digestion by rCED-3 (Fig. 4B, lanes 13–15). These data indicated that the CED-3 recognition site must reside in an 81 amino acid stretch (414–494). Interestingly, this small region contains none of the consensus DXXD motifs within IFG-1. While CED-3 is known to recognize the DXXD motif similarly to caspase-3, recent reports have shown that non-classical sites are efficiently utilized by CED-3 due to the relative promiscuity of the enzyme for residues in the P4-P2 positions [28], [29]. A search for such non-conventional caspase processing sites with a P1 aspartate but variation among the P4-P2 amino acids revealed six potential aspartic acids including QLAD^415^, FGLD^419^, RVSD^427^, TTTD^456^, QQRD^467^, and SSKD^494^. To determine which of these might be the major cleavage site, rCED-3 was incubated with radiolabeled IFG-1 1–35, 420–686. This small (36 kDa) substrate lacks most of the N- and C-termini of IFG-1 p170 allowing for more accurate characterization of the central region and its cleavage products (Fig. 4A and 4C). It also encodes only four of the six non-canonical sites. Upon rCED-3 cleavage, a single product was detected that migrated at approximately 30 kDa (Fig. 4C, lane 6). The product co-migrated with the 30 kDa fragment generated from digested IFG 1–686, indicating it must be a C-terminal product. Again the cleavage was caspase-specific as evidenced by similar processing by human caspase-3 and inhibition by z-VAD-fmk (Fig. 4C, lanes 7 and 8). Based on predicted product sizes, it was determined that the P1 aspartate residue was most proximal to the fusion point of IFG 1–35, 420–686. Two tetrapeptide sequences, RSVD^427^ and TTTD^456^were identified as the only motifs whose positions are consistent with a product of 30 kDa. Curiously, these results point to a CED-3 cleavage site that corresponds to none of the predicted consensus sites.

### The major caspase cleavage site in IFG-1 p170 is Asp456

**Figure 5:**
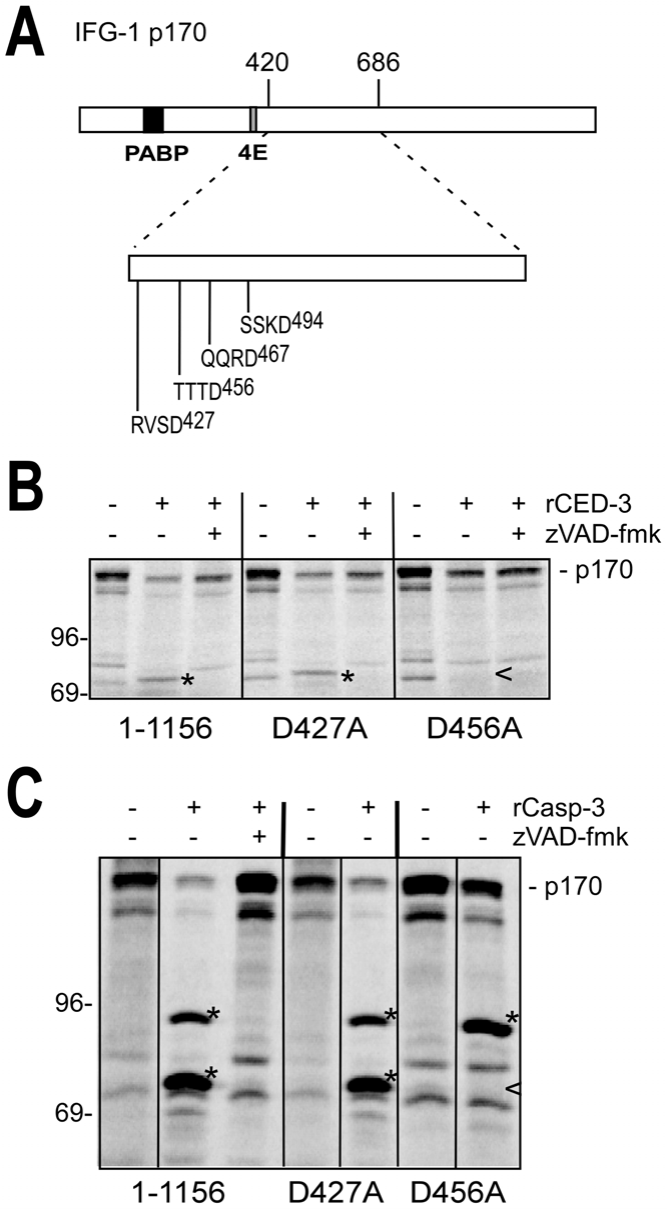
CED-3 cleaves IFG-1 p170 at Aspartate 456. (A) Diagram of full length IFG-1 depicting the rCED-3 cleavage region between amino acids 420 and 686. No canonical caspase 3 recognition sites (DXXD) are present in this region. The positions of the non-canonical caspase cleavage sites (XXXD) are shown. (B) and (C) Aspartates 427 and 456 were mutated to alanines in the context of full length IFG-1 (1–1156). Radiolabeled IFG-1 (1–1156) was incubated with either (B) *C. elegans* rCED-3 or (C) human rCasp-3, resolved by SDS-PAGE, and visualized by phosphorimaging. The (*) indicates processed fragments and a carrot (<) represents the absence of a processed fragment. IFG-1 D456A was resistant to cleavage by rCED-3, and abolished one of the two cleavages by rCasp-3.

Truncation mapping of IFG-1 cleavage suggested that the major processing event occurs at either of two non-canonical sites, RVSD^427^ or TTTD^456^ (Fig. 5A). Unique among eIF4Gs cleaved by caspase-3, the two cleavage motifs in IFG-1 p170 are both located downstream of the predicted eIF4E binding site. To determine which of these sites is used, we performed site-directed mutagenesis to independently change the aspartates to alanines. CED-3 is the only known physiologically relevant caspase for apoptotic cleavage in *C. elegans*, so we tested whether the site-directed mutant IFG-1 D456A was resistant to the worm rCED-3 enzyme and its human homolog. *In vitro* assays of full length IFG-1 (1–1156) D427A and D456A mutants using recombinant *C. elegans* CED-3 (Fig. 5B) and human caspase-3 (Fig. 5C) was then performed and extent of processing assayed by SDS-PAGE. rCED-3 efficiently digested IFG-1 D427A yielding the characteristic 80 kDa C-terminal fragments previously observed from wild type IFG-1. Thus susceptibility of IFG-1 D427A to the worm caspase indicated that RVSD^427^ is not a cleavage site. However, rCED-3 was unable to cleave IFG-1 when TTTD^456^ was mutated to TTTA^456^, as evidenced by the absence of an 80 kDa cleavage fragment (Fig. 5B). These findings confirm that IFG-1 p170 contains a single primary site for CED-3 proteolysis at TTTD^456^. Human caspase-3 likewise digested IFG-1 D427A similarly to wild type IFG-1, but produced fragments of both 95 kDa and 80 kDa (Fig. 5C). IFG-1 D456A, however, was partially resistant to processing, yielding only a single resolved 95 kDa fragment. The absence of the C-terminal 80 kDa fragment indicated that the mutated site prevented cleavage at the major IFG-1 p170 site, TTTD^456^. Interestingly, the secondary 95 kDa product was enriched during cleavage of D456A indicating that this site is more efficiently utilized by human caspase-3 in the absence of the primary processing site. Unlike human caspase-3, rCED-3 showed no cleavage of the secondary site in the context of the full length IFG-1 protein, again suggesting that the worm caspase has a higher specificity for primary single site in worm IFG-1. Collectively these results confirm the non-canonical motif TTTD^456^, which separates the cap-associating N-terminus from the ribosome-binding C-terminus, as the only CED-3 processing site in IFG-1 p170.

### Cleavage of IFG-1 p170 closely mimics native p130 start

**Figure 6:**
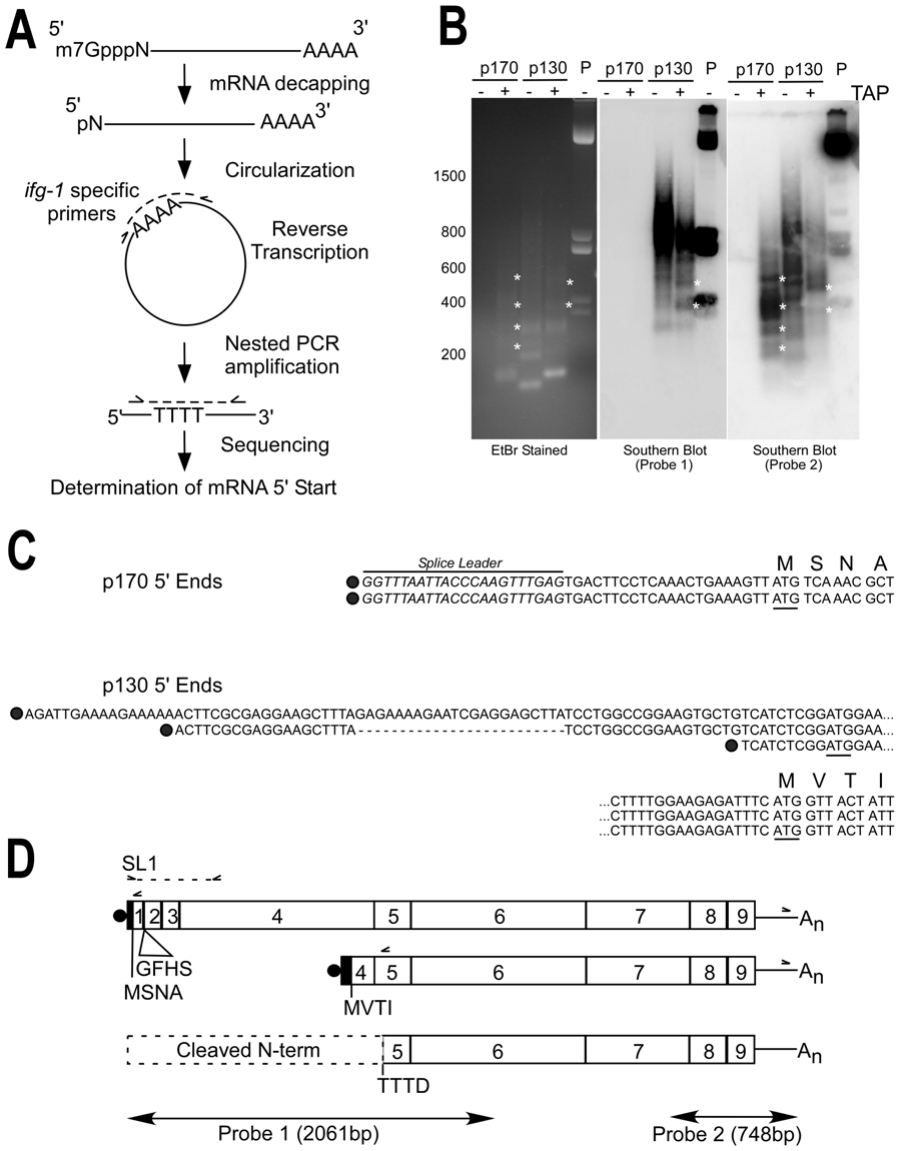
CED-3 cleavage site is immediately downstream of p130 5′ start. (A) Schematic cRT-PCR strategy. *C. elegans* wildtype (N2) total RNA was isolated and messenger RNAs decapped using TAP to generate free 5′-phosphates. Circularization of mRNA was performed by T4 RNA ligase followed by cDNA synthesis by reverse transcription across the ligated junction, and then nested PCR, subcloning and DNA sequencing [51], [52], [53]. Solid lines represent the body of the mRNA; methylated cap, decapped first nucleotide and poly(A) tail are shown. Dashed lines represent cDNA synthesis and PCR amplification. Primer sets used for RT-PCR and nested PCR synthesis are represented by carrots. cRT-PCR using gene-specific primers that flank the *ifg-1* 3′ UTR and either exon 1 (p170) or exon 5 (p130) were used to identify *ifg-1* start sites for both mRNA types (see D). (B) Southern blot analysis of *ifg-1* RNA ligase-mediated products. Capped (TAP +) and uncapped (TAP -) cRT-PCR products were separated by 1.7% gel electrophoresis and stained with ethidium bromide (EtBr) or Southern blotted with either a 5′ end (Probe 1) or 3′ end (Probe 2) *ifg-1* antisense probe. Plasmid (P) containing the *ifg-1* open reading frame was digested with restriction enzymes and loaded as a positive control. Unique, capped mRNA-derived *ifg-1* products are indicated by (*). All short p170 mRNA 5′ end products failed to hybridize to a 5′ end probe that lacked overlap with the amplified region (Probe 1). (C) Sequence alignment of 5′ ends determined for *ifg-1* p170 and p130 mRNAs are shown. Dark circles represent mRNA cap sites. Predicted translation initiation codons (ATG) are underlined and the first four amino acids of the in-frame translation are shown. The *C. elegans* SL1 trans-splice leader is also shown (italics). The two independent clones isolated for p170 mRNA were identical, *trans-*spliced, and corresponded to the strongest probe-2-hybridizing product in (B). The three independent clones isolated for p130 mRNA had divergent starts and were not trans-spliced. Dashed lines indicate alternative *cis-*spliced junction in p130 mRNA, and the ellipsis (…) indicates continuation of sequence on the lines below. (D) Schematic relationship between mRNAs encoding p170 *ifg-1* mRNA, p130 *ifg-1* mRNA, and the site of CED-3 cleavage. Gene specific primers (carrots) in exon 1 (p170), exon 5 (p130), and the *ifg-1* 3′ UTR used for cRT-PCR amplification are shown, as well as the SL1 and exon 4 primers used to verify the *ifg-1* p170 mRNA structure by direct RT-PCR. The regions of hybridization for both *ifg-1* probes used in (B) are also depicted. The first four amino acids encoded by the p170 and p130 open reading frames are indicated. RT-PCR also detected a minor p170 mRNA that encodes four additional amino acids (GFHS) from alternative splice of exon 2.

CED-3-mediated cleavage of IFG-1 p170 at TTTD^456^ generates a C-terminal product that lacks the predicted eIF4E site and thus lacks the ability to associate with mRNA caps. Our previous study showed that the shorter IFG-1 p130 isoform also lacks cap-associating domains. Based on the mapped position for cleavage of IFG-1 p170, it was conceivable that CED-3 processes p170 into a cap-independent fragment nearly identical to native IFG-1 p130 that may further promote cap-independent protein synthesis during apoptosis. To determine whether native IFG-1 p130 encodes a similar protein to the CED-3-cleaved IFG-1 p170, we precisely mapped the 5′ start site for the mRNA encoding the short isoform. Northern and RNase protection analysis previously showed that the mRNA likely begins near the 3′ end of exon 4 [23]. Ligase-mediated mRNA circularization and reverse transcription polymerase chain reaction (cRT-PCR) were used to precisely sequence the 5′ ends of both *ifg-1* p170 and p130 mRNAs (Fig. 6A). Gel electrophoresis followed by ethidium staining showed that in the absence of tobacco acid pyrophosphatase (TAP, cap still present), which removes m7GTP mRNA caps, no p170-derived RT-PCR products were detected (Fig. 6B, EtBr Stained, Lane 1). This indicated that all p170 mRNAs were capped since circularization and ligation were not possible. Upon addition of TAP, however, RT-PCR products ranging from 120–500 bp were observed (Fig. 6B EtBr Stained, Lane 2). To determine if these cDNA fragments corresponded to *ifg-1* sequences, gels were Southern blotted using a ^32^P-labeled probe complementary to *ifg-1* exons 7–9. Hybridization of the *ifg-1* 3′ probe to TAP-treated p170 products identified several bands that corresponded to the ethidium-stained products (Fig. 6B, Probe 2). The pool of TAP-derived p170 products were subsequently subcloned and sequenced. An alignment of sequenced clones revealed identical mRNA 5′ structures. Each contained an SL1 trans-spliced leader followed by 23 nt of untranslated region (UTR) and the translation start site (ATG) (Fig. 6C). Additional RT-PCR using a primer encoding spliced leader SL1 and another complementary to a sequence upstream in exon 4 yielded one major product (not shown) that was also subcloned and sequenced (Fig. 6D). All three results confirm that the mRNA encoding IFG-1 p170 is capped and SL1 trans-spliced.

Our earlier characterization of p130 mRNA showed that the transcriptional start site was located at the 3′ end of exon 4 which would encode an N-terminally truncated IFG-1 protein [23]. To determine the exact position of the mRNA start site, cRT-PCR was performed, using a set of primers located in the *ifg-1* 3′ UTR and just inside exon 5 downstream of putative p130 start sites based on our previous mapping and characterized ESTs. Unlike p170, there were numerous p130 derived PCR products from TAP-untreated (no cap removal) total RNA observed by ethidium stain. Amplified products in non-decapped samples indicate either non-specific PCR artifacts or the presence of a sub-population of p130 mRNAs that may exist naturally uncapped. cDNA synthesis of TAP-treated p130 mRNA, however, showed a slightly modified distribution (Fig. 6B, EtBr, Lane 4). Of these, only a single 270 bp band was found in both TAP-treated and untreated products (Fig. 6B, EtBr, lane 4). While the amplification of a similar product from both treatments suggests that there are both capped and uncapped p130 mRNAs, we focused on those unique to the TAP decapping conditions to be certain we were considering only *bona fide* mRNAs. Southern blot analysis using the *ifg-1* 3′ probe detected hybridization to TAP-derived p130 products of 370 bp and 470 bp that did not overlap with sizes from TAP-untreated samples (Fig. 6B, Probe 2). Clones of these capped p130 mRNAs were sequenced and showed 5′ UTRs that varied in length from 9–84 bp upstream of an out-of-frame ATG (bp 1194–1196), followed 20 nt downstream by an in-frame ATG that begins coding at MVTI (Fig. 6C). Such non-coding short upstream open reading frames are common in short isoforms of eIF4G [2], [6], [34]. The predicted size of *ifg-1* p130 mRNA using these mapped 5′ ends is approximately 2300 bp, which matches well with our previous northern and RNase protection characterization of p130 mRNA [23]. Unlike p170 mRNA, a splice leader was never observed in any p130 clones, and is consistent with the lack of RT-PCR derived products using SL1/SL2-specific primers. The diversity of 5′ ends indicates that p130 transcripts are initiated by an alternative internal promoter within *ifg-1*. One such mRNA also contained a splice junction that removed a 22 nt intron within exon 4 (Fig. 6C). Collectively the data indicate that *ifg-1* p170 mRNAs have a homogeneous start, short 5′ UTR and are SL1-trans-spliced, whereas *ifg-1* p130 transcripts have 5′ end heterogeneity, longer UTRs, and are not trans-spliced (Fig. 6D).

### IFG-1 p130 is also a substrate for CED-3

**Figure 7:**
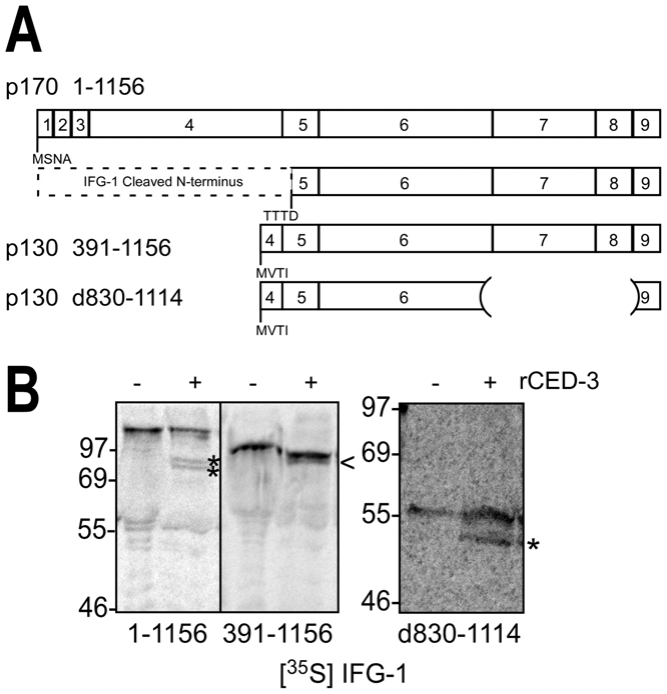
CED-3 cleaves IFG-1 p130 *in vitro*. (A) Schematic diagram depicting the mapped CED-3 cleavage site and translation starts in full length IFG-1 p170 (1–1156), p130 (391–1156) and an internally truncated version of p130 (d830–1114). Numbered boxes correspond to protein encoding exons. (B) Radiolabeled IFG 1–1156, 391–1156 and d830–1114 proteins were incubated with rCED-3 (+) as described above, resolved by 12% or 4-20% SDS-PAGE, and visualized by phosphorimaging. Control reactions (−) were incubated with bovine serum albumin. Cleaved fragments are represented by asterisks (*). A product migrating just under IFG p130 (391–1156; <) was indistinguishable from an internal start site product, preventing conclusive determination of cleavage. However, cleavage of an internally truncated IFG p130 (d830–1114) produced a product that was better resolved and was conclusive.

Most striking from the above RNA mapping is that the proposed initiating methionine (M^391^) for IFG-1 p130 is just 65 amino acids upstream of the caspase (CED-3) cleavage site that identified in IFG-1 p170 (TTTD^456^; Fig. 6D). This indicates that the C-terminal CED-3 cleavage product is a similar cap-independent IFG-1 protein to that translated from p130 *ifg-1* mRNA. Given the proximity of the proposed p130 translation start (MVTI) and the cleavage site, we attempted to determine if p130 was still cleaved by rCED-3 (Fig. 7A). We subcloned and expressed a [^35^S]methionine-labeled IFG-1 p130 (391–1156) in RRL. Radiolabeled IFG-1 p130 was then treated with rCED-3 and gave rise to a very closely migrating smaller product that could just be resolved by SDS gel electrophoresis (Fig. 7B). However, because we frequently observed a closely migrating smaller product in untreated p130 due to a second translation start (data not shown), we could not definitively demonstrate cleavage by rCED-3. To conclusively show that this site in p130 was recognized and cleaved, we generated an internal deletion construct (IFG p130 d830–1114) whose cleavage product could be clearly resolved migrating several kDa smaller than the parent peptide (55 kDa), consistent with loss of the N-terminal 65 amino acids (Fig. 7B). Thus, although not apparent from western blots of native protein, IFG-1 p130 is also processed by CED-3.

### IFG-1 isoforms act upstream of CED-3 and CED-4 during germ cell apoptosis

**Figure 8:**
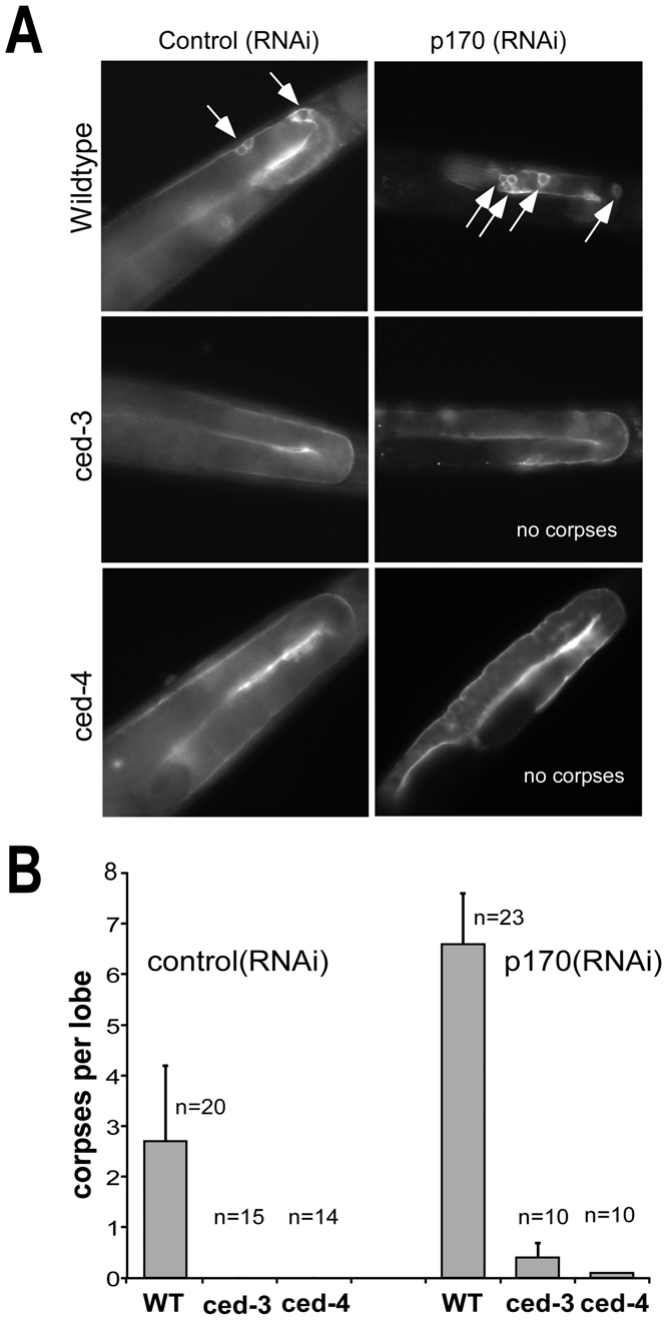
IFG-1 p170 depletion induces apoptosis that requires the caspase and apoptosome. (A) Fluorescence images of Control (RNAi) and p170 (RNAi)-treated adults from wild type, *ced-3* (n2452) and *ced-4* (n1162) strains expressing the apoptotic marker CED-1::GFP. Worms were fed E. coli expressing the dsRNA corresponding to each RNAi target, and the gonads of young adult F1 offspring (similarly fed) were analyzed by fluorescence microscopy. White arrows indicate GFP positive apoptotic germ cell corpses. (B) Bar graph depicting quantitative comparison of germ cell apoptosis induced by *ifg-1* p170 mRNA depletion based on number of GFP-decorated corpses per lobe of the gonad. ‘n’ refers to number of adult gonad lobes in which corpses were counted. Error bars display S.E.M.

The cell death abnormal (*ced*) pathway includes several genes responsible for both the execution and elimination of cells [19]. Data presented in this study identify IFG-1 p170 as a substrate for the main effector caspase in *C. elegans*, CED-3. The catalytic interaction suggests that a caspase-dependent mechanism may modulate protein synthesis during apoptosis in nematodes. But the means by which cap-independent IFG-1 p130 induces apoptosis in the germ line is still unclear. To determine whether cap-independent IFG-1 activity is merely a downstream byproduct of the caspase cascade, or might be an upstream initiator of apoptosis in germ cells, we used epistatic analysis to determine which event comes first. Strong loss-of-function (lf) mutations in the core apoptotic genes, *ced-3* (n2452) and *ced-4* (n1162) have been shown to cause increased survival of germ cells in the gonad [30], [35], [36]. Deficient worms were crossed with a *ced-1:gfp* marker strain to assay for dying cells by GFP decoration. IFG-1 p170 isoform was subsequently depleted (relative to p130) using p170-specific RNAi to disrupt cap-dependent translation as we have previously reported [23]. Loss of p170 should effectively mimic caspase-3 cleavage, whether or not caspase activity is present. Following depletion, the extent of apoptosis in the germ line was assayed by observing the number of oocyte corpses decorated with CED-1:GFP. Wild type worms treated with control RNAi sequences (unrelated to *ifg-1*) exhibited two to five corpses indicating normal levels of physiological apoptosis. These corpses appeared as small fluorescent orbs in the characterized region of germ cell death (Fig. 8A). Knockdown of IFG-1 p170 in wild type worms induced clusters of germ cell corpses (∼2.4 fold; Fig. 8A and 8B) consistent with previously reported observations [23]. Surprisingly, worms lacking the executioner caspase [*ced-3*(lf)] exhibited no appreciable germ cell apoptosis despite the depletion of IFG-1 p170 (Fig. 8B). If the disruption of protein synthesis caused by CED-3 cleavage of IFG-1 were merely part of the dying process, corpses might be expected to appear when cap-dependent synthesis was disrupted by other means. This was not the case. Thus, the germ cell death caused by *ifg-1* (RNAi) is suppressed by the loss of caspase activity.

Interestingly, p170 (RNAi)-treated *ced-3* worms were able to produce both mature oocytes and fertilized embryos, in contrast to p170 (RNAi)-treated wild-type worms that possessed very few mature oocytes and virtually no fertilized embryos ([23] and data not shown). This observation suggests that when apoptosis is prevented, oocytes depleted of cap-dependent translation can still mature and be fertilized. These data further imply that CED-3 prevents oocyte survival for fertilization in the absence of cap-dependent translation.

We next addressed whether the apoptosome protein Apaf-1/CED-4 was required for IFG-1-mediated apoptosis. Depletion of IFG-1 p170 by RNAi was conducted in *ced-4*(lf) worms. Again, germ cell apoptosis was not observed in RNAi-treated *ced-4*(lf) worms, suggesting that *ifg-1*-mediated apoptosis requires the Apaf-1 homolog (Fig. 8A and 8B). Furthermore, several embryos were detected in the uterus of *ced-4*(lf) worms lacking p170 (data not shown), a similar phenotype to *p170*(RNAi) *ced-3*(lf). The combined RNAi/genetic data show that *ifg-1* p170 and p130 function upstream of both *ced-3* and *ced-4*. Germ cell apoptosis as a consequence of IFG-1 p170 depletion is dependent on the apoptosome; both its assembly (Apaf-1) and subsequent proteolytic activation (caspase). The order of these events is consistent with our previous observation that p170 depletion induced CED-4 synthesis prior to the morphological changes preceding oocyte shrinkage and engulfment [23]. We propose that protein synthesis mechanism and eIF4G isoforms play an active role in the decision-making process between growth and apoptosis in germ cells.

## Discussion

The decision to commit cellular suicide, like other events requiring changes in gene expression, depends heavily on new protein synthesis. The initial phases of apoptosis induced by somatic cell damage are marked by a substantial quantitative decrease in protein synthesis activity [37], [38], [39]. However, the translation of select apoptotic mRNAs (Apaf-1, DAP-5/p97, Bcl-2, and XIAP) escape this inhibition. Rather, the *de novo* synthesis of these proteins is sequentially upregulated during cell death because they contain internal ribosome entry sites (IRES) within their 5′ UTRs that promote robust translation initiation in a cap-independent manner. Recent data suggests that IRES selection modulates protein expression in a biphasic manner during apoptosis depending on the severity of insult. For example, cells initially respond to UV irradiation by synthesizing the X-linked Inhibitor of Apoptosis (XIAP) protein via cap independent translation to prevent immediate activation of the caspase cascade [13]. Likewise, Bcl-2 mRNA contains an IRES that is induced as an early response to stress when cap-dependent synthesis is compromised [40]. Both proteins have anti-apoptotic functions. Such responses suggest cap-independent translation facilitates initial attempts by the cell to recover from transient injuries. However XIAP synthesis is not enhanced by chronic apoptotic stress [6]. Instead, prolonged exposure to severe apoptotic conditions causes irreversibly damaged cells to switch their synthetic output. Now pro-apoptotic proteins such as Apaf-1 and p97 are synthesized in a caspase-dependent manner by IRES-driven translation, pushing the cell to a “point of no return” [6], [13], [41]. Thus, the organization and mode of protein synthetic events play a critical role in the timely response needed to both protect cells during distress and ultimately cause their necessary demise.

**Figure 9:**
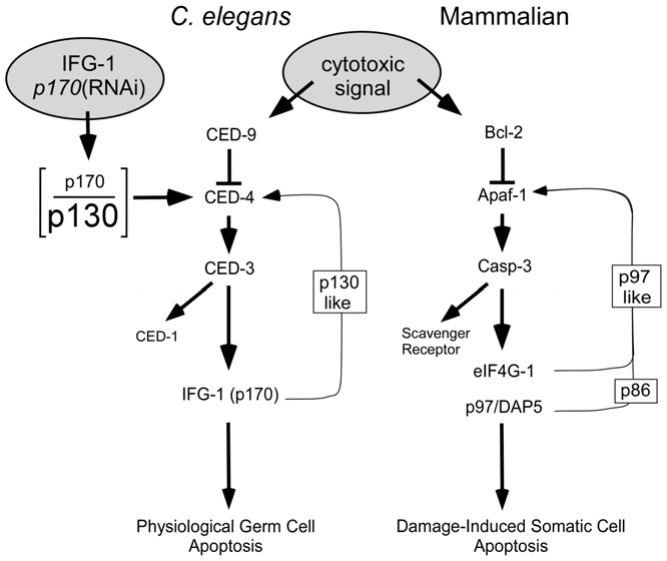
Model for protein synthesis regulation (through eIF4G) of somatic and germ cell apoptosis. In human somatic undergoing apoptosis, eIF4Gs (including p97) are cleaved by caspase-3. The cleavage products support a positive feedback loop that commits the cell to irreversible programmed suicide. This loop is initiated by an insult that disrupts the anti-apoptotic functions of Bcl-2, promoting the formation of apoptosomes from Apaf-1 subunits and sequential activation of a caspase cascade. Caspase-3 causes disruption of the cap-dependent translation initiation complex and further promotes the cap-independent synthesis of Apaf-1 and other apoptotic proteins via internal ribosome entry sites (IRESs) in their mRNAs. In *C. elegans* germ cells, natural variation or genetic disruption [*p170*(RNAi)] of the cellular balance (depicted as the p170/p130 ratio) between cap-dependent and cap-independent IFG-1 isoforms can initiate apoptosis. Data presented here demonstrate that the caspase cascade is affected upstream (likely at CED-4) by eIF4G cleavage. Additionally, IFG-1 p170 is a direct substrate for CED-3, and processed into a p130-like fragment. Accumulation of the cap-independent fragment may then mimic the function of native p130 to secondarily enhance the synthesis of CED-4 protein. Therefore, a similar positive feedback loop may guide the natural selection of oocytes for apoptosis as a function of their translational activity.

Apaf-1 induces the activation of caspases as a signal promoting the apoptotic cascade [42]. The caspase-mediated cleavage of the translation initiation factor eIF4GI and p97 promote Apaf-1 translation via an IRES element. The stable cleaved products, called M-FAG and p86, respectively, retain the highly conserved MIF4G core domain that still binds eIF4A and eIF3 to mediate ribosome recruitment, association with mRNA, and cap-independent/IRES-driven translation [8], [26], [27], [43]. Cleavage also abrogates association between eIF4G and 7-methylguanosine 5′ cap of mRNAs, decreasing the utilization of highly cap-dependent mRNAs. Synthesis of pro-apoptotic proteins is enhanced, while that of cell cycle and growth proteins (e.g. VEGF, c-Myc, cyclin D1), which rely heavily on cap-mediated translation, is hindered. Cleavage of eIF4G thus directs a shift in translation initiation mechanism enacted as switch from a cap-dependent to cap-independent mode. The switch creates a positive feedback loop in the caspase cascade through Apaf-1 synthesis (right side of Fig. 9). Therefore, protein synthetic regulation reinforces a cell's commitment to die while at the same time halting cell cycle progression.

Curiously, the physiological signals that trigger normal developmental cell deaths (e.g. in embryos and the germ line) are less well understood because no external insult is required [25]. Clearly, germ line stem cells that successfully negotiate oocyte or sperm development readily utilize protein synthetic mechanisms to carry out growth and differentiation [44], [45], [46]. In all differentiating cell types, including germ cells, one natural potential fate is apoptotic death. We have previously shown that germ cell deaths during oocyte development can likewise use a protein synthetic mechanism to trigger their suicidal fate [23]. Cell deaths induced as a result of disrupting the balance between cap-dependent and cap-independent translation lead to *de novo* apoptosome (CED-4) formation. Using RNAi to deplete the long eIF4G isoform (IFG-1 p170), the remaining short isoform (IFG-1 p130) can promote only cap-independent translation. As such, IFG-1 p170 appears to protect oocytes from programmed cell death through cap-dependent synthesis. It stands to reason that germ cells, which cease dividing and silence gene transcription during differentiation, should select between developmental alternatives (gamete formation or suicide) by protein synthetic changes. These adaptive changes can therefore determine alternate or even sequential fate decisions. Here we demonstrate that eIF4G isoforms, and the translation initiation mechanism changes they promote, have a primary role in deciding those fates.

In worms, as in humans, initiating apoptosis promotes the activation of caspases and the digestion of several structural and signaling proteins in the cytoplasm, among them all isoforms of eIF4G. While the proteolysis of eIF4Gs has been extensively characterized, little is known about the fate of each cleavage product or their role in apoptotic progression. Here we show that *C. elegans* translation factor eIF4G (IFG-1) is a substrate for the cysteine protease CED-3, and that loss of p170 integrity activates germ cell apoptosis in a caspase-and apoptosome-dependent manner. Although we cannot yet conclude that p170 cleavage is essential for oocyte apoptosis to occur, it has clearly been identified as sufficient to trigger germ cell death. The cleavage products generated by CED-3 indicate specific targeting of the cap-dependent function of the long IFG-1 isoform. We demonstrate that both human caspase-3 and CED-3, (the worm executioner caspase) cleave the worm eIF4G, IFG-1 p170 into discrete N- and C-terminal fragments. These results show that the *C. elegans* IFG-1 is a relevant target during germ cell apoptosis.

The convincing demonstration of CED-3 cleavage of *C. elegans* substrates *in vivo* has remained elusive to many labs. It is difficult to generate a significant homogeneous population of apoptosing cells relative to the healthy cells remaining in the whole animal [28]. We have succeeded in showing the *in vivo* cleavage of IFG-1 p170 by disrupting the function of Bcl-2/CED-9 and inducing extensive temperature-sensitive germ line apoptosis. We also showed less robust *ex vivo* cleavage of native cap-dependent IFG-1 p170 by using recombinant CED-3 and human caspase-3. Furthermore, we delineated the exact site of cleavage by CED-3 using *in vitro* cleavage of recombinant, radiolabeled IFG-1 that was more efficient. Remarkably, cleavage was mapped to a region devoid of caspase consensus (DXXD) motifs. Site-directed mutations revealed that a single non-conventional sequence at TTTD^456^ was targeted to generate discrete C-terminal and N-terminal fragments. Other studies have reported similar recognition of non-consensus caspase sites in worm and *Drosophila* substrates [28], [29], [47]. Additionally, we found that the CED-3 cleavage site in IFG-1 p170 was just 65 amino acids downstream of the predicted start site (AUG/Met) for the naturally occurring cap-independent IFG-1 isoform, p130. Therefore, as a natural consequence of apoptotic signaling, CED-3 is utilized to process IFG-1 p170 into a p130-like protein that promotes cap independent translation and the cell death fate (Fig. 9).

The apoptotic cascade is highly conserved between humans and nematodes, but the signals that induce the pathway during physiological, developmental death are poorly understood [25]. Until now, protein synthesis changes during apoptosis have been viewed more as an “effect” rather than a “cause” [10], [12]. The consequences of dramatic changes in protein synthesis have been interpreted as part of the actual dying process. But no previous evidence suggested that protein synthesis regulation or eIF4G cleavage was a signal to initiate apoptosis. At best, IRES activation (e.g. Apaf-1, DAP-5) was seen to reinforce or “hasten” the death [6], [13]. As such, “downstream” protein synthesis changes might be expected to lead to cell death even without the initiating signals (e.g. Apaf-1 and caspase-3). This is clearly not the case in germ cell apoptosis. Our study suggests that the *C. elegans* eIF4G acts as an upstream activator of caspase-mediated apoptosis, rather than a consequence, during the early development of germ cells. Enhanced cap-independent synthesis was sufficient to induce germ cell corpses, but only if Apaf-1/CED-4 and caspase-3/CED-3 were present (Fig. 8). Despite the ability of CED-3 to cleave IFG-1 p170, the caspase appears to require a signal from IFG-1 isoforms (through CED-4) in order for apoptosis to be initiated by this pathway. Increasing the proportion of cap-independent IFG-1 p130 isoform causes concomitant synthesis of death-associated factors like CED-4 [23] leading to subsequent activation of CED-3. This puts protein synthesis mechanism in the unexpected position of being an initiating signal for germ cell apoptosis through the standard apoptosomal protease cascade (Fig. 9). Such changes are not simply physiological outcomes in cells that have already decided to die. Our findings refute the notion that translation factors are simply inadvertent victims in a crumbling cell. Instead it appears that eIF4G (IFG-1) plays a more decisive role in coordinating apoptotic events than has previously been realized.

Finally, apoptosing cells use protein synthesis regulation to “finish the job”. Enhanced caspase activity is expected to cause further proteolytic processing of eIF4Gs resulting in more cap-independent synthesis to reinforce the apoptotic cascade. Modification of the protein synthetic mechanism during mammalian apoptosis changes the pool of mRNAs being translated. The utilization of death-associated mRNAs containing IRES elements is required for rapid changes in growth conditions during apoptosis (reviewed in [13]). However, little is known about how apoptotic mRNAs are utilized during germ cell death in worms. Recent screens have identified two zinc-finger RNA binding proteins, GLA-1 and GLA-3, that play a prominent role in the suppression of germ cell death [48], [49]. Such proteins are known to repress the translation of stored mRNAs in oocytes by binding specific elements in the 3′ UTR. GLA proteins may take part in the translational control of apoptosis that we have outlined for IFG-1. Alternatively, specific regulation of *ced-4* mRNA itself may be at work in germ cells. Two isoforms of CED-4/Apaf-1 derived from alternative splicing have been described that differ in function during apoptosis. CED-4S has been shown to be pro-apoptotic, while CED-4L is anti-apoptotic [36]. The splicing event is regulated by serine-arginine-rich kinase, SPK-1 in somatic apoptosis during neural development [50], but it is unclear if this event also occurs in the germ line. Because developing oocytes tend to accumulate fully spliced mRNAs for subsequent translational regulation, we might speculate that mRNA utilization plays a more important role for these two isoforms than splicing during germ cell apoptosis. A shift between IFG-1 isoforms might, for example, facilitate preferential CED-4S synthesis in oocytes destined to die. Alternatively, relative activities of IFG-1 p170 and p130 could mobilize mRNAs regulated by GLA proteins to reverse potential translational repression. Though the details of the exact role of eIF4G in germ cell apoptosis remain to be uncovered, the addition of *ifg-1* to the cell death pathway genes suggests that a highly conserved regulatory event during apoptosis is modulated by protein synthesis mechanism.

A remarkable cascade of molecular events allows cells to control their proliferation, coordinate differentiation, and minimize damage through an organized cell death program. Apoptosis is necessary for the disassembly and removal of cells that either disrupt neighboring cell function or are unnecessary for the architecture of tissues [1], [28]. It has become clear that the demolition process is not a static series of cataclysmic steps without purpose, but instead a rational alternative. The integrity of IFG-1 p170 is thus overtly maintained in certain germ cells to prevent entry into the apoptotic cascade and allow them to complete oogenesis. Others are selected for removal. While the circuitry of protein synthesis regulation during germ cell apoptosis requires further investigation, it is clear that a fine-tuned balance of translational control is necessary for the developmental competence of early germ cells and their maturation to egg and sperm.

## Materials and Methods

### Strains

*C. elegans* strain N2 var. Bristol and mutant strains were grown at 20^o^C on normal growth medium (NGM) plates with *E. coli* strain OP50 or HT115 containing plasmids that synthesize double-stranded RNA (dsRNA) unless otherwise noted (Brenner, 1974). Strains *ced-3* (n2452), *ced-4* (n1162) and *ced-9* (n1653) *mab-5* (mu114) obtained from the Caenorhabditis Genetics Center were crossed into *ced-1::gfp*-expressing MD701 (*bcls39v [P_lim-7_ ced-1::gfp* and *lin 15(+)]*) which was kindly provided by Dr. Barbara Conradt (Dartmouth University). *ced-3* and *ced-4* homozygous lines expressing the transgene were obtained by self crossing progeny carrying the deletion or displaying phenotype, and picking individuals to fresh seeded NGM plates for observation at 20°C or 25°C. The mutant alleles of the resulting stable homozygous strains, KX84 (*ced-3* loss-of-function), KX87 (*ced-4* loss-of-function) were monitored for the absence of GFP-positive germ line corpses in untreated worms. KX110 (*ced-9* loss-of-function) was scored for GFP-positive germ line corpses following temperature shift to 25°C. The *ced-3* genomic deletion was also verified by PCR using the primers, forward 5′-AGTTCACCGTGACAGCGTCTCTTC-3′ and reverse 5′-CGATTACGACTTGAACTGTATCCGA-3′ (wild type allele) or reverse 5′-CATTGCAAATCTCGTTTACACAAGA-3′ (mutant allele). A minimum of 20 gonads were analyzed for each experiment. The WormBase designation for the single eIF4G gene (*ifg-1*) in *C. elegans* is WBGene00002066.

### Plasmid Constructions

For recombinant CED-3 expression, the caspase catalytic region (CED-3^3221–503^) was amplified from N2 total RNA using forward 5′-GCCCATATGCACCACCACCACCACCACGTCGATGCACCAACCATAAGCC-3′, which includes a 6X His tag, and reverse 5′-GCCAAGCTTTTAGACGGCAGAGTTTCGTGCTT-3′ primers. The PCR fragment was subcloned into pGEX4T-1(GE Healthcare) to generate pGSTH6CED-3^221–503^. Templates for *in vitro* transcription and translation of IFG-1 protein and truncations were in pBlueScript SK (Stratagene). cDNA encoding IFG-1 115–1156 (nt 366–3494) was obtained from Dr. Yugi Kohara (National Institute of Genetics, Japan)**.** To create full length *ifg-1* cDNAs (nt 1–3494), extended 5′ ends were amplified by RT-PCR from N2 RNA using forward primers corresponding to the 5′ end of the longest expressed sequence tag (EST), forward 5′-CGCGGATCCATGTCAAACGCTGTTAGTAGGG-3′, or the SL1 spliced leader, forward 5′-GGTTTAATTACCCAAGTTTGAG-3, and reverse 5′-CGCATCGTTTTGATATCCAATCCG-3′, subcloned into pCRII TOPO TA (Invitrogen), and sequenced. The verified 5′ end corresponding to the longest known *ifg-1* EST was subcloned via BamHI and NheI into the existing cDNA to create pSKifg-1 Long that encodes full length IFG-1 1–1156. Similarly, pSKifg-1 p130mvti was generated by PCR amplification of nt1194–1264 and subcloning via BamHI and NheI. Upon sequencing a single base mutation was discovered in the third codon (ACT to ATT) that caused a missense substitution of Thr for Ile. This change did not prevent CED-3 cleavage. The mutation was not present in pSKifg-1 p130 d830–1114. Template plasmids for *in vitro* translated IFG-1 substrates for caspase cleavage assays were constructed from pSKifg-1 Long or pSKifg-1 p130mvti. Restriction digestion and re-ligation of pSKifg-1 Long generated the following truncation constructs: IFG 1-414 (nt 24–1265), IFG 1–686 (nt 24–2083), IFG d278–494 (nt 24–857, 1509–3494), IFG 1–35, 420–686 (nt 24–128, 1281–2083), p130 IFG 391–1156 (nt 1194–3494), and IFG p130 d830–1114 (nt 1194–2510, 3366–3494). PCR-mediated site-directed mutagenesis was carried out to create IFG D427A using primers, forward 5′-GCTCAGCTAGCTGATTTCGGATTGGATATCAAAACGATGCGACTTTCTGCTAATAAG-3′ and reverse 5′-AAGAGAGCGAGATGTTTTTG-3′. IFG D456A was generated using complementary oligos containing the directed mutation to generate two *ifg-1* fragments with overlapping ends. The double primer sets included forward 5′-ATGTCAAACGCTGTTAGTAGGG-3′ and reverse 5′-TTCCAGCTGTGGTTGTTCTTC-3′ for the 5′ amplicon and forward 5′-AAGAACAACCACAGCTGGAAC-3′ and reverse 5′-TCACCAGTAACGGTCCACAA-3′ for the 3′ amplicon. The resulting fragments were then mixed and amplified further using nested primers, forward 5′-TCAGACACAACCACCACTAC-3′ and reverse 5′-AAGAGAGCGAGATGTTTTTG-3′. The final PCR products for both IFG D427A and IFG D456A were digested and subcloned into pSKifg-1 Long to create caspase resistant constructs. All constructs were verified by DNA sequencing (Iowa State DNA Sequencing Facility).

### Expression and purification of recombinant CED-3

Recombinant *C. elegans* CED-3 (rCED-3) was expressed in *E. coli* DH5α cells as previously described (Taylor, 2007). Proteins were affinity purified using Ni-NTA sepharose (Invitrogen) and eluted in CED-3 reaction buffer (50 mM HEPES, pH 7.4, 150 mM NaCl, 0.5 mM sucrose, 5% glycerol) with 250 mM imidazole. Elutions were supplemented with DTT and glycerol to a final concentration of 2 mM and 20% respectively. Protein concentrations were determined by Bradford method using a bovine serum albumin (BSA) standard curve. Fractions containing recombinant H_6_CED-3^221–503^ were verified by immunoblotting using an anti-His6 monoclonal antibody (Genscript). Caspase activity was quantified using the colorimetric substrate Ac-DEVD-pNA (Promega) as described by the manufacturer. 10 µl of pooled peak fractions was incubated with the substrate (0.2 mM) at 37°C and absorbance at 405 nm monitored over a 4 h period. Activity was calculated using a pNA standard curve.

### rCasp-3/rCED-3 cleavage assays and immunoblotting

Recombinant enzymes were pre-incubated at 37°C for 30 min to 1 h prior to all cleavage assays. Protein substrates were labeled with [^35^S]-methionine (Perkin Elmer Life Sciences) using TnT T3 coupled reticulocyte lysate system (Promega). Ten microliter reactions were prepared containing 0.5 µg of plasmid DNA and 0.8 µl of [^35^S]methionine (10 mCi/mL). One microliter of *in vitro* synthesized product was incubated with 10 µl of recombinant CED-3 at 37°C for 2 h. Control reactions substituted with 10 µl of non-catalytic BSA were prepared in CED-3 reaction buffer. Reactions were stopped by adding equal volumes of 4X SDS buffer. Samples were loaded on Novex 4–20% Tris-Glycine Midi Gel (Invitrogen), resolved by electrophoresis, dried, and radioactivity analyzed by phosphorimaging using Typhoon 9410 scanner at the ECU PhIFI Core Facility. Cell free *C. elegans* protein extract *ex vivo* reactions were performed using frozen N2 worm pellets ground in the presence of liquid nitrogen and 2X extract buffer (100 mM HEPES pH 7.4, 0.2% CHAPS, 100 mM NaCl, 2 mM PMSF, 50 µg/mL leupeptin, 10 mM DTT) supplemented with a Halt protease inhibitor cocktail (Thermo Scientific). Insoluble material was pelleted 30 min at 14000 x g at 4°C. rCED-3 (10 µl) was immediately incubated with soluble worm extract for 2 h at 37°C. Both *in vitro* and *ex vivo* reactions were also incubated with either the pan-caspase inhibitor z-VAD-fmk (Promega) or caspase-3-specific inhibitor, Ac-DEVD-CHO (Sigma). *Ex vivo* reactions or worm extracts were resolved by 6% or 8% SDS-PAGE gels and immunoblotted using IFG-1 central domain antibody or N-terminal antipeptide antibody as previously described [23].

### RNA isolation, RNA analysis, and cRT-PCR

Total RNA purification was performed using the Trizol method as previously described [23]. cRT-PCR was conducted as described by Mullen and Marzluff, 2008 [51]. Approximately 70 µg of total N2 RNA was treated with 25 units of RQ1 DNase (Promega) in a 100 µl total volume and incubated for 30 min at 37°C. RNA was extracted with a 1∶1 volume of phenol:chloroform:isoamyl alcohol and precipitated from 70% ethanol. RNA was resuspended in 20 µl of RNase free H_2_O. Purified total RNA (10 µg) was treated with 2.5 units of tobacco acid pyrophosphatase (Epicentre) in a 20 µl reaction and incubated for 1 h at 37°C. Following a second phenol:chloroform extraction and ethanol precipitation, nucleic acids were resuspended in 10 µl of H_2_O. Four micrograms of either TAP-treated (+) or –untreated (−) RNA was incubated with 20 units of T4 RNA ligase (Epicentre) and 20 units of RNasin (Promega) in 400 µl reaction for 16 h at 16°C. A final extraction and precipitation was performed in the presence of 10 µg of glycogen and the circularized RNA resuspended in 12 µl H_2_O. One microgram of ligated RNA was then amplified by RT-PCR using Superscript One Step RT-PCR with Platinum Taq (Invitrogen) using primary *ifg-1* primers, forward 5′-TCGAGCTATTTGACGGGCAA-3′ and reverse 5′-TGTGGTTGGTTGTATTGTT-3′ (*ifg-1 p170*) or reverse 5′-GCGCATCGTTGGAGGATACA-3′ (*ifg-1 p130*). Nested PCR was performed using 1 µl of cDNA with GoTaq DNA polymerase (Promega) and primers, forward 5′-CCTACCGATTTTATGTCTTATTGGG-3′ and reverse 5′-CCCTACTAACAGCGTTTGACAT-3′ (*ifg-1 p170*) or reverse 5′-ATCTCTTTGCTGCTGACCGCCA-3′ (*ifg-1 p130*). The products were subcloned directly into pCRII TOPO TA vector (Invitrogen) and *ifg-1* positive clones sequenced. For Southern blotting, PCR products were resolved on 1.7% agarose gel and transferred to NitroPlus 2000 membrane (MSI Separations) and hybridized overnight at 58°C with the designated probes. After stringently washing the membrane with 2X SSC at 60°C, hybridization signals were analyzed with Typhoon 9410 imager and ImageQuant TL software. The antisense Riboprobes used were transcribed from the plasmids containing nt 24–2083 and nt 3092–3494 of *ifg-1* full length cDNA as described [23]. As a control, plasmid pF170 containing full length *ifg-1* open reading frame was digested with EcoRV and EcoRI to generate multiple fragments for comparison against cRT-PCR products.

### RNA interference and Microscopy

RNAi by feeding was performed as previously described [23]. Briefly, knockdown of IFG-1 p170 utilized dsRNA transcribed from 398 bp (nt 24–479) of the *ifg-1* cDNA expressed in HT115 (DE3) seeded onto NGM plates containing 1 mM isopropyl β-D-1-thiogalactopyranoside (IPTG) and 100 µg/mL ampicillin. Four to six L4 hermaphrodite worms of the designated strains were transferred to the fed RNAi plates and subsequently incubated at 22°C for several days. Adult hermaphrodites from the F1 offspring were immobilized in 30 nM sodium azide in M9 buffer and germ cell corpses expressing CED-1:GFP examined using a Zeiss Axiovert 200 M microscope equipped with an Axiocam MRM2 CCD camera, FITC/GFP optics, and F-fluar 40X objectives as described [23]. Germ line apoptosis was induced in *ced-9ts* (n1653) by shifting synchronous L3 larvae to 25°C for 48 h, followed by fluorescence microscopy or extract preparation as described above.
